# Nickel-mediated modulation of soil urease activity and nitrogen uptake in maize cultivated on Egyptian calcareous soil

**DOI:** 10.1186/s12870-026-10077-9

**Published:** 2026-11-02

**Authors:** Ahmed M. Mahdy, Nieven O. Fathi

**Affiliations:** 1 Department of Soil and Water, College of Agriculture, Alexandria University, Alexandria, Egypt; 2 Soil Salinity & Alkalinity Laboratory, Ministry of Agriculture, Alexandria, Egypt

**Keywords:** Maize, Nickel, N utilization, Urease activity, Crude protein, Calcareous soil, Nutrient interaction, Egyptian soils

## Abstract

Nitrogen (N) utilization in maize production is frequently constrained by nutrient interactions and the physicochemical properties of calcareous soils. Nickel (Ni), an essential micronutrient and structural component of urease, plays a critical role in nitrogen metabolism; however, its beneficial and toxic thresholds under Egyptian calcareous soil conditions remain poorly understood. Therefore, a greenhouse pot experiment was conducted to investigate the interactive effects of Ni and N on maize (*Zea mays* L., single hybrid 10 white pioneer) growth, nitrogen uptake, soil urease activity, and crude protein accumulation in an Egyptian calcareous soil. Four nitrogen levels (0, 30, 60, and 120 mg N kg⁻¹ soil as urea) and five nickel levels (0, 5, 10, 15, and 20 mg Ni kg⁻¹ soil as NiCl₂·6 H₂O) were arranged in a factorial completely randomized design with four replications. Soil urease activity, dry matter yield, nitrogen uptake, nickel uptake, and crude protein content were determined at harvest. The results demonstrated highly significant effects (*P* < 0.001) of N, Ni, and their interaction on all measured parameters. Dry matter yield and nitrogen uptake increased progressively with increasing N fertilization. Nickel exhibited **a** biphasic dose response, where moderate concentrations (5–10 mg kg⁻¹) enhanced plant performance, whereas higher concentrations (15–20 mg kg⁻¹) reduced growth due to toxicity. The highest urease activity (78 ± 5.1 µg NH₄⁺-N g⁻¹ soil h⁻¹) and crude protein content (37.06 ± 1.62%) were obtained under the combined application of 120 mg N kg⁻¹ and 10 mg Ni kg⁻¹. Quadratic regression models accurately described maize dry matter responses to Ni application (R² = 0.90–0.99), confirming an optimum Ni range before the onset of toxicity. Excessive Ni concentrations significantly reduced biomass production, urease activity, and crude protein accumulation through physiological disturbances associated with metal toxicity. Overall, the study demonstrates that balanced Ni supplementation can substantially improve nitrogen utilization efficiency and nutritional quality of maize grown in Egyptian calcareous soils, whereas excessive Ni application is detrimental. The combined application of 120 mg N kg⁻¹ soil and 10 mg Ni kg⁻¹ soil appears to be the most effective treatment under the conditions of this pot experiment.

## Introduction

Nitrogen (N) deficiency is a major constraint to maize production because N is essential for chlorophyll formation, photosynthesis, protein synthesis, and biomass accumulation. Inadequate N supply reduces leaf growth, chlorophyll content, plant biomass, and ultimately grain yield. The problem is particularly important in low-fertility and calcareous soils, where low organic matter and limited nutrient availability can restrict N uptake. Therefore, improving N availability and nitrogen-use efficiency is essential for achieving high maize productivity while minimizing fertilizer losses and environmental impacts. In maize (*Zea mays* L.), adequate nitrogen nutrition is essential for photosynthesis, biomass accumulation, kernel development, and overall crop yield. Nitrogen availability strongly influences carbon assimilation, root architecture, leaf area development, and grain filling, making it one of the primary determinants of maize productivity [1, 2]. However, nitrogen utilization remains relatively low worldwide because a substantial proportion of applied nitrogen is lost through volatilization, leaching, denitrification, and runoff, resulting in economic losses and environmental concerns [3]. Furthermore, NUE is often constrained by soil physicochemical properties and interactions with micronutrients, particularly in calcareous soils, which are characterized by high pH, elevated calcium carbonate content, and reduced nutrient availability. These conditions can limit nutrient uptake and alter nutrient interactions, thereby reducing crop productivity and nutrient use efficiency [1]. Recent advances in maize nutrition research have highlighted the importance of understanding the genetic, physiological, and soil-related mechanisms regulating nitrogen uptake, assimilation, and remobilization to develop sustainable nutrient management strategies and improve maize performance under diverse environmental conditions [2].

Urease is an enzyme produced by soil microorganisms, plants, and some soil fauna. It catalyzes the hydrolysis of urea into ammonia (NH₃) and carbon dioxide (CO₂). Measuring urease activity is commonly used to assess soil biological activity, nitrogen cycling, and soil fertility [4].

Nickel (Ni) is recognized as an essential micronutrient for higher plants because it is a structural component of the enzyme urease, which catalyzes the hydrolysis of urea into ammonium and therefore plays a crucial role in nitrogen metabolism [5, 6]. Nickel is required only in trace amounts, and adequate concentrations contribute to improved nitrogen utilization, enhanced seed germination, enzyme activation, and overall crop performance. Recent studies have further demonstrated that nickel participates in several physiological processes, including nitrogen assimilation, antioxidant regulation, and maintenance of cellular homeostasis [7, 8]. However, similar to other micronutrients, excessive nickel accumulation becomes toxic and adversely affects plant growth, metabolism, and productivity. Elevated nickel concentrations can induce oxidative stress, disrupt nutrient balance, impair photosynthetic machinery, alter root development, and reduce biomass production, ultimately compromising crop performance [7]. Therefore, maintaining nickel concentrations within an optimal range is essential to balance its beneficial nutritional role against its potential phytotoxic effects.

Nickel toxicity has been associated with numerous physiological and biochemical disturbances, including inhibition of photosynthesis and respiration, impairment of mineral nutrition, disruption of enzymatic activities, reduction in chlorophyll content, restriction of water uptake, alteration of assimilate transport, and induction of oxidative stress [9–12]. Recent studies have further demonstrated that elevated nickel concentrations impair photosynthetic efficiency, disrupt antioxidant defense systems, alter gene expression, induce nutrient imbalance, and reduce biomass accumulation in maize plants [13]. In addition, excessive nickel exposure negatively affects chlorophyll biosynthesis, photosystem II efficiency, membrane stability, and redox homeostasis through excessive production of reactive oxygen species (ROS), ultimately suppressing plant growth and productivity [14, 15]. Molecular studies have also revealed that nickel toxicity can alter the expression of genes involved in metal transport, detoxification pathways, antioxidant metabolism, and stress signaling networks, highlighting the complexity of plant adaptive responses to nickel stress [16].

The effects of nickel on nitrogen metabolism are particularly important because nitrogen assimilation depends on several nickel-sensitive physiological processes. Excess nickel may interfere with root development, nitrogen transporter activity, nitrate reduction, amino acid biosynthesis, and protein formation, thereby disrupting the coordination between nitrogen uptake and assimilation [7, 17]. Such disturbances ultimately reduce nitrogen use efficiency and plant productivity. Recent studies have shown that abiotic stresses affecting nitrogen metabolism substantially alter carbon–nitrogen balance, amino acid synthesis, and nutrient use efficiency in maize, emphasizing the need for integrated studies on nitrogen–micronutrient interactions [17]. Furthermore, nickel stress has been shown to modify antioxidant metabolism, nutrient homeostasis, and stress-responsive gene expression in maize, thereby exacerbating the adverse effects on nitrogen metabolism and growth performance [13]. Emerging evidence also suggests that nickel toxicity directly influences nitrogen metabolism and amino acid profiles in maize, highlighting the need to better understand nickel-induced changes in nitrogen utilization under different soil conditions [18].

Calcareous soils occupy substantial agricultural areas in Egypt and are characterized by high calcium carbonate (CaCO₃) content, alkaline pH, low organic matter, and reduced nutrient availability. These properties strongly influence micronutrient mobility, nutrient interactions, and crop productivity by decreasing the availability of several essential nutrients, particularly nitrogen, phosphorus, iron, zinc, and manganese [19]. Maize cultivation in Egyptian calcareous soils often faces challenges related to poor nutrient use efficiency, making a better understanding of nickel–nitrogen interactions increasingly important for sustainable crop production. Recent studies in Egypt have emphasized that improving nutrient management strategies, including balanced fertilization, micronutrient management, and soil amendments, can significantly enhance nutrient availability, nitrogen use efficiency, and maize productivity under calcareous soil conditions [20, 21]. Although considerable progress has been made in understanding nickel phytotoxicity, most studies have primarily focused on oxidative stress responses, antioxidant defense mechanisms, molecular tolerance pathways, and remediation strategies [7, 16]. Comparatively little attention has been given to the direct influence of nickel on nitrogen uptake, nitrogen assimilation, and nutrient use efficiency in maize, particularly under Egyptian calcareous soil conditions. Different studies have shown that nickel stress can alter nitrogen metabolism, amino acid composition, and nutrient homeostasis in maize; however, these investigations have largely been conducted under controlled conditions without considering the unique physicochemical characteristics of calcareous soils [18]. Despite the increasing interest in Ni nutrition and toxicity, an important research gap remains regarding the interactive relationship between Ni and N in maize grown in calcareous soils. Previous Ni × N studies have generally examined either Ni toxicity, individual nutrient responses, or N metabolism under controlled hydroponic or non-calcareous soil conditions. Consequently, there is still limited information on whether N availability modifies the response of maize to increasing Ni concentrations under the high-pH and CaCO₃-rich conditions characteristic of Egyptian calcareous soils. In addition, relatively few studies have simultaneously evaluated a broad range of Ni and N levels to distinguish their individual effects from their interactive effects on maize growth, nutrient accumulation, and N-use performance. *Addressing this gap* is important because soil properties can substantially modify Ni availability and nutrient interactions, and therefore responses observed under hydroponic or other soil conditions may not necessarily apply to calcareous agricultural soils [18–21].Therefore, the present study was designed to investigate the individual and interactive effects of Ni and N supply on maize grown in calcareous soil. Specifically, the objectives were to **(i)** evaluate the effects of increasing Ni and N levels on maize growth and biomass production, **(ii)** assess their effects on N uptake and accumulation and related nutrient-use performance, and **(iii)** determine whether N availability modifies maize responses to increasing Ni concentrations under calcareous soil conditions. The study hypothesized that **(1)** increasing N availability would enhance maize growth, N accumulation, and nutrient-use performance; **(2)** Ni would have a concentration-dependent effect, with appropriate Ni availability potentially supporting plant N metabolism, whereas excessive Ni would impair growth and nutrient balance; and **(3)** the effects of Ni would depend on the level of N supply, resulting in a significant N × Ni interaction. Collectively, this study aims to provide new insights into N–Ni interactions in maize under calcareous soil conditions and to contribute to the development of more balanced nutrient-management strategies for maize production in Egyptian soils.

## Materials and methods

### Soil characterization

A calcareous soil (*Typic calciorthids*) was collected from the surface layer (0–30 cm depth) of Borg Al-Arab region, Alexandria Governorate, Egypt. The soil was air-dried, gently crushed, and passed through a 2-mm sieve prior to laboratory analyses. Soil electrical conductivity (EC) was measured in saturated soil paste extract following the standard procedure described by Richards [22]. Soil pH was determined in a 1:2.5 soil-to-water suspension according to Richards [22]. Organic matter content was estimated using the dichromate oxidation method described by Nelson and Sommers [23]. Cation exchange capacity (CEC) was determined using 1 N sodium acetate extraction according to Rhoades [24]. Particle size distribution was analyzed using the hydrometer method of Day [25]. Calcium carbonate content was determined using the calcimeter method described by Nelson [26].

Available nitrogen was determined using the Kjeldahl digestion method described by Bremner and Mulvaney [27], while available phosphorus was extracted using 0.5 M sodium bicarbonate according to Olsen and Sommers [28]. Available potassium was extracted using 1 N ammonium acetate following the procedure of Knudsen et al. [29]. These analytical procedures are widely accepted in soil fertility, nutrient availability, and soil contamination studies [30, 31]. Selected physicochemical properties of the soil are presented in Table 1.

### Experimental design and treatments

A controlled pot experiment was conducted under greenhouse conditions to investigate the interactive effects of nitrogen (N) and nickel (Ni) on the growth and physiological performance of maize (*Zea mays* L.). A commercial maize hybrid, Single Hybrid 10 White Pioneer, was used as the test crop. Seeds were obtained from the Maize Research Department, Field Crops Research Institute, Agricultural Research Center (ARC), Giza, Egypt. Uniform, healthy, and disease-free seeds with high germination capacity were selected before sowing.

The experiment was established using a 5 × 4 factorial arrangement consisting of five Ni levels (0, 5, 10, 15, and 20 mg Ni kg⁻¹ soil) and four N levels (0, 30, 60, and 120 mg N kg⁻¹ soil). Nickel was supplied as nickel chloride hexahydrate (NiCl₂·6 H₂O), whereas nitrogen was supplied as urea. The treatments were arranged in a completely randomized design (CRD) with four independent replicates, resulting in 20 treatment combinations and 80 experimental pots. Each pot represented one experimental unit. The selected N rates provided a gradient from N deficiency to relatively high N availability, while the Ni treatments allowed evaluation of maize responses to increasing Ni supply and its interaction with N nutrition. The treatment ranges and pot-experiment approach were selected with reference to previous studies investigating N nutrition and Ni responses in maize and other cereal crops. Polyethylene pots were filled with 5 kg of processed soil. Before filling the pots, the soil was thoroughly homogenized to ensure uniformity among experimental units. The required amounts of Ni and N were calculated on a soil-mass basis and applied according to the respective treatment levels. Nickel treatments were incorporated uniformly into the soil to ensure relatively homogeneous distribution within each pot. Nitrogen was applied as urea in two equal applications, with 50% applied at sowing and the remaining 50% applied 30 days after sowing. Splitting the N application was intended to improve synchronization between N availability and crop demand and to reduce potential N losses compared with a single application. To avoid deficiencies of nutrients other than the experimental variables, all pots received a uniform basal application of phosphorus, potassium, and selected micronutrients. These nutrients were supplied at rates equivalent to 50 mg P, 63 mg K, 10 mg Zn, 10 mg Fe, 5 mg Cu, and 5 mg Mn kg⁻¹ soil. The same amounts were applied to all treatments so that differences in plant responses could primarily be attributed to the imposed N and Ni treatments. The basal nutrients were incorporated uniformly into the soil before sowing.

Two maize seeds were sown in each pot at a uniform depth. After germination and establishment, seedlings were inspected and thinned to one healthy and uniform plant per pot to maintain the same plant density across all experimental units. Pots were randomly positioned within the greenhouse, and their positions were periodically randomized to minimize possible positional effects associated with differences in light, temperature, or airflow within the greenhouse. Soil moisture was maintained at approximately 70% of field capacity throughout the experimental period. Distilled water was used for irrigation, and the water requirement was adjusted according to changes in soil moisture and plant water consumption. All treatments were maintained under the same moisture regime to prevent water availability from becoming an additional experimental factor. Greenhouse conditions were monitored throughout the experiment, and the plants were allowed to grow for 10 weeks before harvest. The factorial design enabled the independent effects of N and Ni to be evaluated as well as their interaction. Thus, the experiment was not designed merely to compare individual N or Ni treatments but specifically to determine whether the plant response to Ni varied according to N availability. The four independent replicates for each treatment combination provided the experimental basis for statistical evaluation of the main effects of N and Ni and their interaction.

### Plant sampling and chemical analysis

At harvest, shoots were collected, washed thoroughly with distilled water to remove adhering soil particles, and oven-dried at 65 °C for 48 h until constant weight was achieved. Dry matter yield was recorded on a dry weight basis. Dried plant samples were ground using a stainless-steel mill for chemical analysis.

For nickel determination, plant material was dry-ashed at 450 °C for 6 h in a muffle furnace. Ash was dissolved in 1:1 HNO₃, diluted to a known volume, and analyzed for Ni concentration using atomic absorption spectrophotometry (AAS) according to Jones and Baker and Amacher [32, 33]. Total nitrogen concentration in plant tissues was determined after acid digestion using the Kjeldahl method [27, 32].

Nutrient uptake (Ni and N) was calculated by multiplying nutrient concentration by the corresponding dry matter yield. This calculation approach is commonly applied in plant nutrition and heavy metal accumulation studies [30, 31].

### Urease activity determination in soil

Urease activity was determined in soil treated and un-treated with nitrogen and nickel levels according to the reaction:

$$\mathrm{CO} (\mathrm{NH}_2)_2 + \mathrm{H2O}\rightarrow 2\mathrm{NH}_3+ \mathrm{CO}_2$$

The ammonia released during incubation is quantified by distillation. Urease activity is then expressed as the amount of NH₄⁺-N released per gram of soil per unit time according to Tabatabai, and Bremner [4].

$${\rm{Urease}}\,{\rm{activity}}\,=\,{\mu}g \,{\rm{NH}}_4 {^{+}} - {\rm{N}}\,{\rm{released}}/{\rm{g}}\,{\rm{soil}} \times {\rm{h}}$$

Soil urease activity was determined by measuring the amount of NH₄⁺-N released following the hydrolysis of urea. Briefly, 5.0 g of air-dried and sieved soil was placed in a 50-mL incubation tube and treated with 1.0 mL of toluene to inhibit microbial proliferation without substantially affecting urease activity. Subsequently, 10 mL of citrate buffer (pH 6.7) and 10 mL of 10% (w/v) urea solution were added to the soil, and the contents were thoroughly mixed. The samples were incubated at 37 °C for 24 h under controlled conditions. After incubation, the soil suspension was extracted with 50 mL of 1 M KCl solution, shaken thoroughly, and filtered. The concentration of NH₄⁺-N released during urea hydrolysis was determined colorimetrically using the indophenol-blue method, with absorbance measured at approximately 578–630 nm against an appropriate reagent blank. A calibration curve prepared using known concentrations of ammonium nitrogen was used to calculate the NH₄⁺-N concentration in the extracts. Urease activity was expressed as µg NH₄⁺-N released g⁻¹ soil h⁻¹, based on the amount of NH₄⁺-N produced during the incubation period. All determinations were performed using appropriate analytical replicates.

### Crude protein estimation (Kjeldahl method)

Organic nitrogen present in plant samples was converted into ammonium sulfate by digestion with concentrated sulfuric acid in the presence of catalysts. During distillation, ammonia was liberated by adding alkali, trapped in boric acid solution, and quantified by titration with standard acid according to the official analytical procedures described by Horwitz and Latimer [34].The measured nitrogen is converted to crude protein. Crude protein (CP) is estimated by determining the total nitrogen (N) content of a sample and multiplying it by a conversion factor (usually 6.25).

$$\mathrm{Crude}\,\mathrm{Protein} \,(\%) =\mathrm{Nitrogen} \,(\%) \times 6.25$$

(The factor 6.25 assumes proteins contain approximately 16% nitrogen.)

### Statistical and mathematical analysis

Data were statistically analyzed using SAS software, version 6 [35]. A two-way analysis of variance (ANOVA) was conducted using **PROC GLM** to evaluate the main effects of the experimental factors and their interaction. When significant differences among treatment means were detected, means were separated using Fisher’s least significant difference (LSD) test at *p* ≤ 0.05. Prior to ANOVA, the assumptions of normality and homogeneity of variance were assessed using the Shapiro–Wilk test and Levene’s test, respectively. Residual plots were also examined to identify potential deviations from normality, unequal variance, or other influential patterns. The data met the assumptions required for ANOVA; therefore, no data transformation was necessary. Results are presented as treatment means, and statistical significance was declared at *P* ≤ 0.05.

To describe the relationship between soil nickel concentration and maize dry matter yield under different nitrogen levels, a second-order polynomial regression model was applied:

${\rm{Yi}}=B_0 +B_1 X_i +B_2 X_i^2$ according to Thabet and Balba [36], 

Where:

- Yi = predicted dry matter yield.
- Xi = actual nickel application rate.
- B0 = intercept.
- B1, B2 = linear and quadratic coefficients.

Ni efficiency** (**E_Xi_**)** was calculated from the fitted quadratic regression equation Yi = B_0_+B_1_X_i_+B_2_X_i_^2^ as E_xi_=B_1_−2B_2_X_i_, where Xi represents each actual Ni application rate used in the experiment (0, 5, 10, 15, and 20 mg Ni kg⁻¹ soil). This expression is the first derivative of the quadratic response function and therefore represents the predicted marginal change in the measured plant response per unit increase in Ni at a specific Ni rate. Accordingly, E_Xi_ has units corresponding to the measured response per mg Ni kg⁻¹ soil and is not a conventional Ni-use efficiency index. The derivative-based approach was employed to characterize how the plant response changed progressively along the Ni dose–response curve and to identify whether the marginal response increased or declined as Ni supply increased. It was therefore used as a complementary dose–response descriptor rather than as a substitute for conventional agronomic efficiency indices such as agronomic efficiency, recovery efficiency, or partial factor productivity. This modeling approach is consistent with recent agronomic response curve analyses used in nutrient optimization and metal toxicity studies [3, 31]. Moreover, quadratic regression analysis was further used to estimate the Ni rate associated with the maximum predicted response at each N level. For each fitted model, the mathematical optimum Ni rate was calculated from the quadratic equation as X = − B1/ (2B2).

## Results and discussions

### Soil characterization

The calcareous soil used in this study exhibits typical physicochemical properties of arid and semi-arid region soils, particularly those widely distributed in Egypt (Table 1).

**Table 1:** Characteristics of Calcareous Soil (Mean ± SD)

Characteristics	Units	Calcareous Soil
EC	dS m⁻¹	2.92 ± 0.18
pH		8.08 ± 0.05
CaCO₃	g kg⁻¹	356.80 ± 12.40
Sand	g kg⁻¹	740.00 ± 18.60
Silt	g kg⁻¹	101.50 ± 6.20
Clay	g kg⁻¹	158.50 ± 7.90
Texture		S.L
O.M†	g kg⁻¹	4.60 ± 0.35
CEC	cmol(+) kg⁻¹	26.00 ± 1.10
Olsen-P	mg kg⁻¹	18.70 ± 1.25
Available-N	mg kg⁻¹	61.00 ± 3.80
Available-K	mg kg⁻¹	301.00 ± 11.50
Available-Ni	mg kg⁻¹	7.17 ± 0.45
WHC¶	g kg⁻¹	470.00 ± 15.00

The relatively high pH (8.08 ± 0.05) confirms the alkaline nature of the soil, which is primarily attributed to the substantial calcium carbonate content (356.80 ± 12.40 g kg⁻¹). Such alkaline conditions are widely reported to reduce the solubility and plant availability of essential nutrients, particularly phosphorus and several micronutrients, through precipitation and adsorption reactions [30]. Recent studies have further emphasized that calcareous soils strongly regulate nutrient speciation and mobility, thereby influencing crop nutrient uptake efficiency and fertilizer response under field conditions [19, 31]. The soil texture is classified as sandy loam (S.L), with a dominant sand fraction (740.00 ± 18.60 g kg⁻¹), followed by clay (158.50 ± 7.90 g kg⁻¹) and silt (101.50 ± 6.20 g kg⁻¹). This coarse texture, combined with low organic matter content (4.60 ± 0.35 g kg⁻¹), indicates weak aggregation, low nutrient retention capacity, and limited water-holding potential. Such conditions are commonly associated with enhanced leaching losses of mobile nutrients, particularly nitrogen, thereby reducing nitrogen use efficiency in crop production systems [1, 3]. The moderate water-holding capacity (470.00 ± 15.00 g kg⁻¹) further reflects the limited moisture retention typically observed in sandy loam calcareous soils under arid environments. The electrical conductivity (2.92 ± 0.18 dS m⁻¹) indicates non-saline to moderately saline conditions. Although not severely saline, such EC levels may still impose mild osmotic stress, especially under limited irrigation regimes. The cation exchange capacity (26.00 ± 1.10 cmol(+) kg⁻¹) reflects moderate nutrient retention potential; however, this value is still constrained by low organic matter and high sand content, which together reduce the soil’s buffering capacity and nutrient-holding efficiency [31]. Available nitrogen (61.00 ± 3.80 mg kg⁻¹) and Olsen phosphorus (18.70 ± 1.25 mg kg⁻¹) indicate moderate fertility status; however, nutrient availability in calcareous soils is often limited by fixation reactions with calcium carbonate and low organic matter inputs. Recent research has shown that such soils exhibit strong nutrient immobilization effects, which directly affect nitrogen transformation processes and fertilizer recovery efficiency [1, 19]. Available potassium (301.00 ± 11.50 mg kg⁻¹) is relatively adequate, reflecting the mineralogical contribution of parent material and low leaching susceptibility for K⁺ compared to nitrogen. Of particular importance to this study is the native nickel concentration (7.17 ± 0.45 mg kg⁻¹), which falls within the normal background range for uncontaminated agricultural soils. However, in calcareous soils, nickel availability is strongly influenced by high pH and calcium carbonate content, which can reduce its solubility through adsorption and precipitation processes. Nevertheless, recent studies have shown that even background levels of nickel can interact with nutrient cycles and become biologically relevant when external nickel inputs are applied [7, 18]. Overall, the soil characteristics indicate a carbonate-rich, alkaline, low-organic-matter system with moderate nutrient constraints and limited buffering capacity. These conditions strongly justify the investigation of nickel–nitrogen interactions, as nutrient mobility, metal availability, and nitrogen use efficiency are expected to be highly sensitive under such calcareous soil environments [3, 31].

### Effect of nickel and nitrogen on dry matter yield of maize

**Fig. 1:**
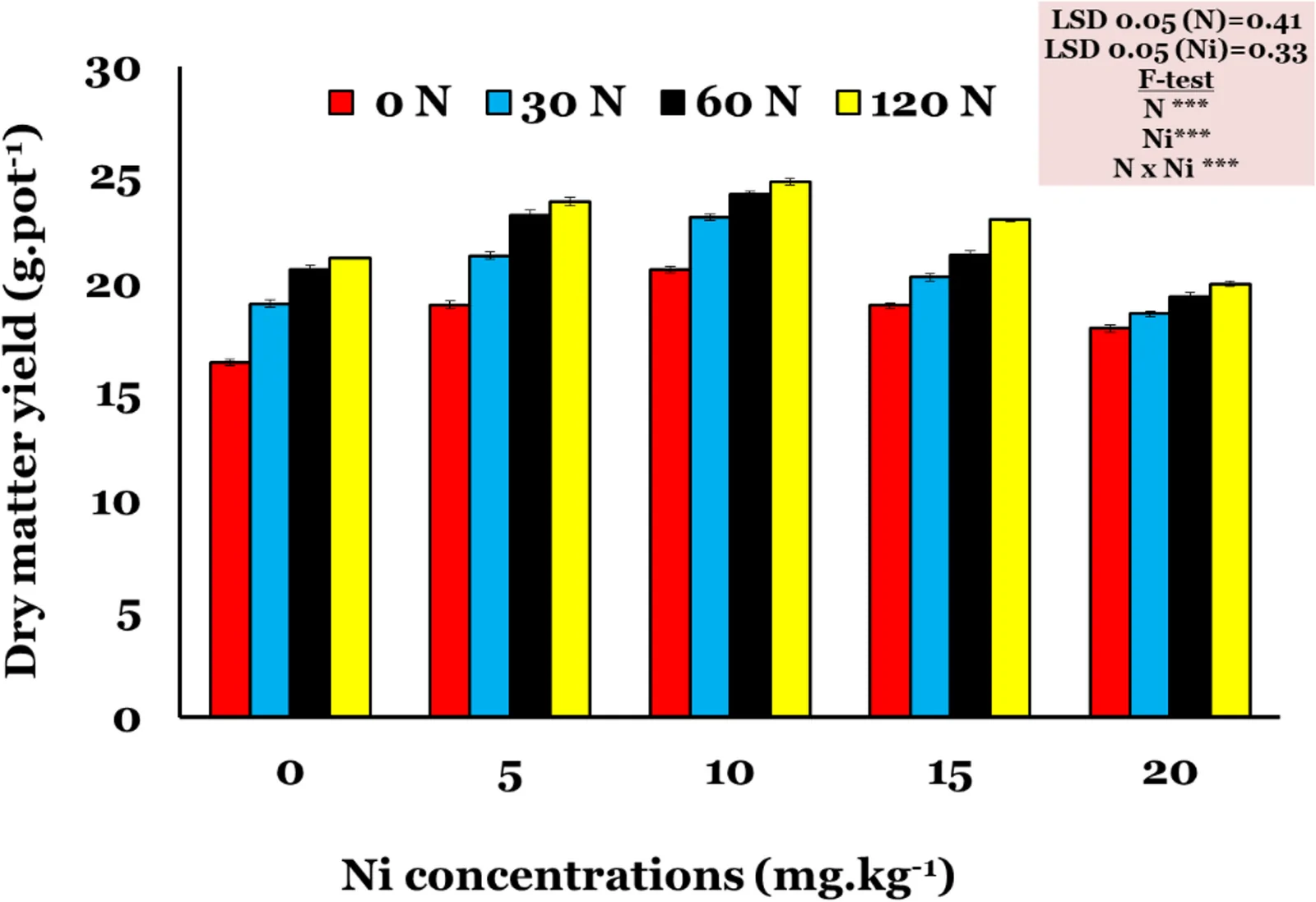
Dry matter yield of maize plant as influenced by an application of N and Ni to soil

The presented figure 1 clearly demonstrates a strong interactive effect between nitrogen (N) fertilization and nickel (Ni) application on the dry matter yield of the tested crop. When interpreted in relation to the LSD values (LSD₀.₀₅ = 0.41 for N and 0.33 for Ni), most of the observed differences among treatment means are statistically significant, confirming that both the main effects of N and Ni, as well as their interaction (N × Ni***), had a real and meaningful influence on biomass production rather than arising from random variation. Similar findings have been widely reported in recent studies emphasizing the central role of nitrogen utilization as a determinant of crop productivity under optimized nutrient management systems [37]. Regarding the main effect of nitrogen, dry matter yield consistently increased with increasing N levels across all Ni treatments. This trend reflects the fundamental role of nitrogen in chlorophyll formation, photosynthetic efficiency, and amino acid and protein biosynthesis. Recent reviews confirm that nitrogen remains the most limiting nutrient in agricultural systems, and its efficient management is essential to sustain biomass production and minimize losses to the environment. The observed increases exceeding the LSD threshold confirm that these responses are statistically robust [38]. In terms of nickel application, a clear dose-dependent response was observed within each nitrogen level. Nickel increased dry matter yield up to an optimum concentration (typically 10–15 mg kg⁻¹), followed by stabilization or decline at higher levels. This response pattern is consistent with recent findings showing that low-dose Ni can significantly improve plant growth and nitrogen metabolism, whereas excessive Ni induces toxicity and growth inhibition. The stimulatory phase reflects a hormetic effect, where low concentrations enhance physiological performance [39]. The significant interaction effect (N × Ni ***) indicates that the response to nickel is strongly dependent on nitrogen availability. The beneficial effects of Ni became more pronounced at higher nitrogen levels, suggesting that Ni enhances nitrogen use efficiency rather than acting independently. This synergistic behavior aligns with recent evidence showing that improving nitrogen utilization requires integrated nutrient strategies that optimize both macro- and micronutrient interactions within plant metabolic systems [37]. Physiologically, this interaction is mainly explained by the role of Ni as a structural component of urease, which catalyzes urea hydrolysis and improves nitrogen assimilation efficiency. Recent studies confirm that Ni directly enhances urease activity, thereby increasing nitrogen recycling and reducing urea accumulation in plant tissues, which ultimately improves biomass production and nitrogen utilization. In addition, Ni contributes indirectly to improved nitrate reduction, amino acid synthesis, and protein formation, strengthening overall nitrogen metabolism efficiency [40]. At higher Ni concentrations (e.g., 20 mg kg⁻¹), the decline in dry matter yield reflects the onset of Ni toxicity. This is consistent with recent literature reporting that excess Ni induces oxidative stress, disrupts enzymatic activity, and impairs chlorophyll stability, ultimately reducing growth performance and biomass accumulation. Such stress responses are commonly associated with elevated reactive oxygen species (ROS) and metabolic imbalance [39]. From a statistical perspective, most differences between Ni levels exceeded the LSD value (0.33), while nitrogen-level differences exceeded LSD₀.₀₅ (0.41), confirming the reliability and significance of the observed trends. The consistency of responses across all treatment combinations further supports the robustness of the dataset [41]. Overall, the results demonstrate that nickel application enhances nitrogen uptake and utilization efficiency, particularly under sufficient nitrogen supply. Recent global analyses of nitrogen utilization confirm that integrated nutrient management strategies significantly improve crop productivity by synchronizing nutrient availability with plant demand. The most favorable concentration under the specific conditions of our pot experiment observed at 10–15 mg kg⁻¹ reflects a balance between beneficial metabolic activation and toxicity thresholds. In conclusion, recent literature strongly supports the present findings, confirming that Ni acts as a functional micronutrient that enhances nitrogen metabolism and biomass production when applied at appropriate concentrations. However, excessive Ni disrupts physiological processes and reduces productivity. Therefore, optimizing the combined application of N and Ni represents an effective strategy for improving nitrogen use efficiency and sustainable crop production. Ni application should be applied cautiously because excessive or repeated Ni inputs may cause soil accumulation, phytotoxicity, and environmental risks. Although 10 mg Ni kg⁻¹ produced favorable responses under the conditions of this pot experiment, it should not be considered a universally recommended rate. Long-term field studies are needed to evaluate Ni accumulation, mobility, crop safety, and potential food-chain risks before practical field-scale recommendations can be made.

### Dry matter modeling

The polynomial quadratic model was employed to describe the relationship between maize dry matter (DM) yield and soil-applied nickel (Ni) concentrations under different nitrogen (N) fertilization levels. The parameters of the model (B₀, B₁, and B₂) were estimated using the least squares method based on experimental pot data. Accordingly, four regression equations were developed to represent the response of maize dry matter yield to Ni application under increasing nitrogen levels:

$$\begin{array}{lll}\mathrm{Y}_{\rm{N}_{o}} &=& 16.546+0.666 \mathrm{X} \\&&-\, 0.030 \mathrm{X}^{2} (\mathrm{R}^2 = 0.90, \mathrm{SE}\,=\,0.70)\end{array}$$

$$\begin{array}{lll}{\rm{YN}}^{30} &=& 19.043+0.703\,{\rm{X}} \\ &&-\, 0.037{\rm{X}}^2 ({\rm{R}}^2\,=\,0.92,\,{\rm{SE}}\,=\,0.72)\end{array}$$

$$\begin{array}{lll}{\rm{YN}}^{60} &=& 20.554+0.704 {\rm{X}} \\ &&-\, 0.040{\rm{X}}^2 ({\rm{R}}^2 = 0.95, {\rm{SE}}\,=\,0.66)\end{array}$$

$$\begin{array}{lll}{\rm{YN}}^{120} &=& 21.234+0.766 {\rm{X}} \\ &&-\, 0.042{\rm{X}}^2 ({\rm{R}}^2 = 0.99, {\rm{SE}}\,=\,0.32)\end{array}$$

The high coefficients of determination (R² = 0.90–0.99) indicate that the quadratic model accurately captures the variability in dry matter yield in response to Ni application under different nitrogen levels. In addition, the relatively low standard errors (SE) confirm a strong agreement between observed and predicted values, demonstrating the reliability and robustness of the fitted models. Similar goodness-of-fit values have been reported in recent studies modeling micronutrient dose–response relationships in cereals under varying nitrogen regimes [42]. The positive linear coefficients (B₁) combined with negative quadratic coefficients (B₂) across all nitrogen levels indicate a typical non-linear hormetic response to Ni application. This suggests that low to moderate Ni concentrations stimulate maize growth, whereas higher concentrations reduce dry matter production due to the onset of toxicity. Such quadratic dose–response behavior is widely documented in recent plant nutrition studies, where micronutrients such as Ni, Zn, and Cu show stimulatory effects at low concentrations and inhibitory effects at higher levels due to oxidative stress and metabolic disruption [43]. A clear nitrogen-dependent shift in Ni responsiveness was also observed. As nitrogen levels increased from 0 to 120 mg N kg⁻¹, both the intercept (B₀) and the slope (B₁) values increased, indicating enhanced baseline productivity and greater responsiveness to Ni application under improved nitrogen nutrition. The highest responsiveness was observed under the highest nitrogen level (Y_N_₁₂₀), suggesting that adequate nitrogen supply enhances the physiological utilization of Ni. This supports the previously observed significant interaction effect (N × Ni ***) and confirms that Ni functions more effectively under conditions of sufficient nitrogen availability. Physiologically, this synergistic interaction can be explained by the essential role of Ni in nitrogen metabolism.

The estimated optimum Ni rates were 11.10 mg kg⁻¹ at N_0_, 9.50 mg kg⁻¹ at N_30_, 8.80 mg kg⁻¹ at N_60_, and 9.12 mg kg⁻¹ at N_120_, respectively. These estimates indicate that the response generally reached its maximum within the low-to-moderate Ni range rather than increasing continuously with Ni supply. However, the calculated optima should be interpreted cautiously because they are model-derived estimates based on the tested Ni concentrations and quadratic functional form. Thus, the relatively high R^2^ and low SE values indicate a good fit to the observed data but, by themselves, do not establish the robustness or universal applicability of the estimated optimum Ni rates. Thus, the mathematical optimum Ni rates range from 8.80 to 11.10 mg Ni kg⁻¹ soil, rather than 10–15 mg kg⁻¹. The estimates are all within the experimentally tested range (0–20 mg kg⁻¹).

The adequacy of the quadratic regression models was evaluated using the coefficient of determination (R^2^), standard error (SE), significance of the linear and quadratic regression coefficients, and residual diagnostics. The significance of the regression coefficients was assessed using the corresponding *P*-values, while residuals were examined for approximate normality, independence, and homogeneity of variance to verify the main assumptions of the regression analysis. Where applicable, lack-of-fit was also considered to determine whether the quadratic model adequately represented the observed response pattern. Although the models showed relatively high R^2^ values (0.90–0.99) and low SE values, these statistics were interpreted together with the regression significance and diagnostic results rather than as independent evidence of model accuracy or robustness. Therefore, the fitted equations are considered to adequately describe the observed Ni dose–response relationships within the experimental range.

Nickel is a key component of the enzyme urease, which catalyzes the hydrolysis of urea into ammonium, thereby enhancing nitrogen availability within plant tissues. Recent research has demonstrated that Ni supplementation improves urease activity, nitrogen recycling efficiency, and protein synthesis, ultimately increasing biomass production and nitrogen use efficiency [7]. Additionally, Ni has been reported to indirectly stimulate nitrate reductase activity and amino acid metabolism, further strengthening nitrogen assimilation pathways in plants.

**Table 2:** Effect of nickel treatments on efficiencies (Ex_i_) of dry matter yield of maize plants grown on soils treated with different rates of nitrogen fertilizer

No. of Ni units	0 *N*	30 *N*	60 *N*	120 *N*
1	3.42	3.55	3.78	4.05
2	1.62	1.78	1.95	2.10
3	-0.25	-0.18	-0.10	-0.05
4	-1.05	-1.28	-1.35	-1.42

The efficiency of Ni treatments (Eₓ_i_) values clearly reflect the dose-dependent and nitrogen-modulated response of maize dry matter yield to nickel application as presented in Table 2. Overall, the table demonstrates a typical biphasic dose response pattern, where low Ni doses stimulate growth, while higher doses induce inhibitory effects. At Ni unit 1, all nitrogen treatments exhibit strong positive Eₓ_i_ values, ranging from 3.42 (0 N) to 4.05 (120 N). This indicates a pronounced stimulatory effect of Ni at low application rates, which is progressively enhanced with increasing nitrogen supply. The highest response under 120 N confirms that adequate nitrogen nutrition amplifies Ni efficiency, likely through improved nitrogen assimilation and enhanced enzymatic activity (particularly urease activation). This aligns well with Fig. 1, where dry matter yield consistently increases with Ni up to the optimum level (around 10 mg kg⁻¹), especially under higher nitrogen regimes. At Ni unit 2, the efficiency remains positive but declines compared to unit 1, ranging from 1.62 to 2.10 across nitrogen levels. This reduction suggests that the system is approaching the threshold of maximum physiological benefit. Although Ni still contributes positively to biomass formation, its marginal efficiency decreases, indicating a transition from strongly stimulatory to moderately beneficial effects. This trend corresponds to the flattening of yield increase observed in Fig. 1 between intermediate Ni levels. At Ni unit 3, Eₓ_i_ values become slightly negative or near zero, ranging from − 0.25 to -0.05, indicating the onset of toxicity or metabolic imbalance. At this stage, Ni begins to exceed the optimal physiological requirement, leading to disruption of enzymatic functions and possible oxidative stress. However, the magnitude of negativity is relatively small, particularly under higher nitrogen levels (60 N and 120 N), suggesting that nitrogen supply partially mitigates Ni-induced stress. This buffering effect is also evident in Fig. 1, where yield reduction at intermediate-high Ni levels is less severe under higher nitrogen treatments. At Ni unit 4, all treatments show clearly negative efficiency values, ranging from − 1.05 to -1.42, indicating strong inhibitory effects of excessive Ni application. This confirms that high Ni concentrations surpass the tolerance threshold of maize plants, leading to reduced dry matter accumulation. The more negative values under higher nitrogen levels suggest that although nitrogen enhances growth potential, it may also intensify metabolic demand, making plants more sensitive once Ni toxicity sets in. This is consistent with Fig. 1, where a decline in dry matter yield is observed at the highest Ni concentration (20 mg kg⁻¹) across all nitrogen levels. Overall, the Table 2 strongly supports the quadratic dose–response behavior observed in Fig. 1, confirming that Ni exhibits a clear stimulatory–optimum–toxic transition. The interaction with nitrogen is highly significant, as higher nitrogen levels consistently increase the magnitude of positive responses at low Ni but also slightly amplify the decline at toxic levels. This indicates that nitrogen improves Ni utilization efficiency but does not fully eliminate toxicity at excessive concentrations. In conclusion, the Eₓ_i_ patterns validate the physiological interpretation of the Ni–N interaction: moderate Ni levels enhance maize productivity under adequate nitrogen supply, while excess Ni leads to metabolic disruption and yield decline. This reinforces the importance of maintaining Ni within an optimal range in integrated nutrient management strategies. The positive efficiency values at lower Ni increments indicate a stimulatory effect on physiological processes such as enzymatic activation and root development. However, the decline in efficiency at higher increments reflects a shift toward toxicity, likely associated with oxidative stress, chlorophyll degradation, and inhibition of metabolic enzymes. Such transitions from beneficial to toxic effects have been widely reported in recent literature on trace metal–plant interactions [43]. Similar optimal ranges have been reported in recent agronomic studies, where low-dose Ni application enhanced crop growth and nitrogen utilization under controlled and field conditions [44]. Recent literature strongly supports these findings, emphasizing that Ni acts as a functional micronutrient involved in nitrogen metabolism and that its agronomic efficiency is maximized under adequate nitrogen supply [42]. In conclusion, the quadratic modeling approach confirms that maize dry matter yield responds non-linearly to Ni application, with clear evidence of a hormetic dose–response pattern. The interaction with nitrogen is highly significant, with optimal performance observed under high nitrogen supply combined with moderate Ni application. These findings reinforce the importance of integrated nutrient management strategies aimed at improving nitrogen use efficiency and sustainable crop productivity under micronutrient-balanced fertilization systems.

### Nitrogen uptake as influenced by Ni & N concentrations in soil

**Fig. 2:**
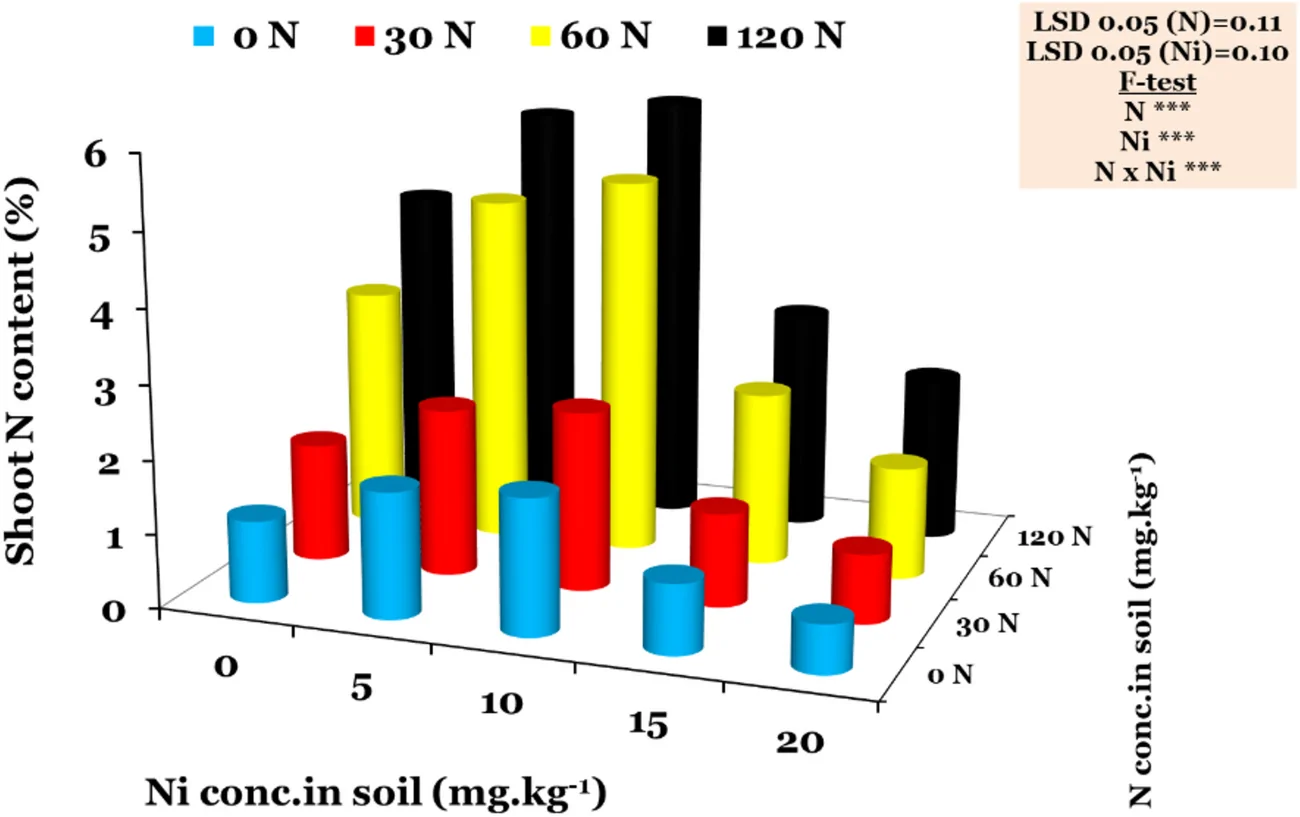
Shoot N content in maize plants as influenced by N and Ni application in soil

The results presented in Fig. 2 demonstrate a clear and significant interaction between nitrogen (N) fertilization and nickel (Ni) concentrations on the shoot nitrogen content response. Overall, increasing nitrogen levels consistently improved the response across all Ni treatments, indicating a strong positive effect of N supply. At the lowest N level (0 N), the values remained low and ranged between 0.66 and 1.85. However, as nitrogen increased to 30 N and 60 N, a progressive increase was observed, while the highest values were recorded at 120 N, reaching up to 5.93 (Fig. 2). This confirms that nitrogen plays a central role in enhancing plant performance, likely through improved photosynthetic activity, protein synthesis, and overall metabolic efficiency, particularly under metal-stressed conditions.

In contrast, nickel exhibited a non-linear, quadratic response pattern. At all nitrogen levels, Ni application at low to moderate concentrations (5–10 mg/kg) enhanced the shoot N response, with the maximum values consistently observed at 10 mg/kg Ni. For example, under 120 N, the shoot nitrogen increased from 4.33 at 0 Ni to 5.65 at 5 mg/kg Ni, reaching a peak of 5.93 at 10 mg/kg Ni (Fig. 2). This indicates that Ni at low concentrations may act as a beneficial micronutrient involved in enzyme activation, particularly urease, thereby enhancing nitrogen metabolism and utilization efficiency. Similar beneficial effects of trace Ni on plant physiological processes have been reported in recent studies e.g., Mustafa et al., [7]; Muzafar et al., [45]. However, further increases in Ni concentration beyond the optimum level (15 and 20 mg/kg) led to a marked decline in the response across all nitrogen levels. At 120 N, values decreased sharply to 3.03 and 2.27 at 15 and 20 mg/kg Ni, respectively (Fig. 2). This reduction reflects the toxic effects of excess Ni, which may disrupt cellular metabolism, inhibit photosynthesis, induce oxidative stress, and interfere with the uptake of essential nutrients such as iron, zinc, and magnesium. These findings are consistent with previous reports indicating that high Ni concentrations impair plant growth and physiological functions due to oxidative damage and enzyme inhibition [42, 46].

The interaction between nitrogen and nickel is also clearly evident from the data. At low nitrogen levels, the beneficial effect of Ni is limited, while at higher nitrogen levels, the positive response to Ni becomes more pronounced, especially at the most favorable concentration under the specific conditions of our pot experiment of 10 mg/kg. The combination of 120 N and 10 mg/kg Ni produced the highest recorded value (5.93), indicating a synergistic interaction between adequate nitrogen supply and optimal nickel availability (Fig. 2). This suggests that nitrogen enhances the plant’s capacity to utilize micronutrients efficiently and may also improve tolerance mechanisms against moderate Ni exposure. Overall, the results indicate that nitrogen fertilization enhances plant performance under nickel treatments, while nickel shows a clear biphasic dose response, being beneficial at low concentrations and toxic at higher levels. The most favorable condition observed in this study was the combination of high nitrogen (120 N) with 10 mg/kg Ni (Fig. 2), which can be considered the most favorable concentration under the specific conditions of our pot experiment for maximizing the measured response. These findings agree with recent literature highlighting the importance of balanced N nutrition in improving micronutrient efficiency and mitigating heavy metal stress in plants [44].

### Ni concentration in soil and plant uptake

The data show a clear and strong main effect of nickel (Ni) concentration on the measured response, alongside a consistent but opposite effect of nitrogen (N) fertilization. Overall, increasing Ni levels from 0 to 20 mg/kg resulted in a pronounced and nearly linear increase in the response at all nitrogen levels. For example, under 0 N, the values increased sharply from 10.17 at 0 Ni to 102.00 at 20 mg/kg Ni. A similar trend was observed under 120 N, where values rose from 7.88 to 87.09 across the same Ni range. This indicates that Ni shoot content or Ni-associated response is strongly dose-dependent, reflecting enhanced shoot content of Ni in plant tissues as external supply increases. In contrast, nitrogen application showed a consistent decreasing effect on the measured response across all Ni concentrations. At 0 Ni, values declined from 10.17 under 0 N to 7.88 under 120 N, while at 20 mg/kg Ni, the response decreased from 102.00 to 87.09 with increasing N.

**Fig. 3:**
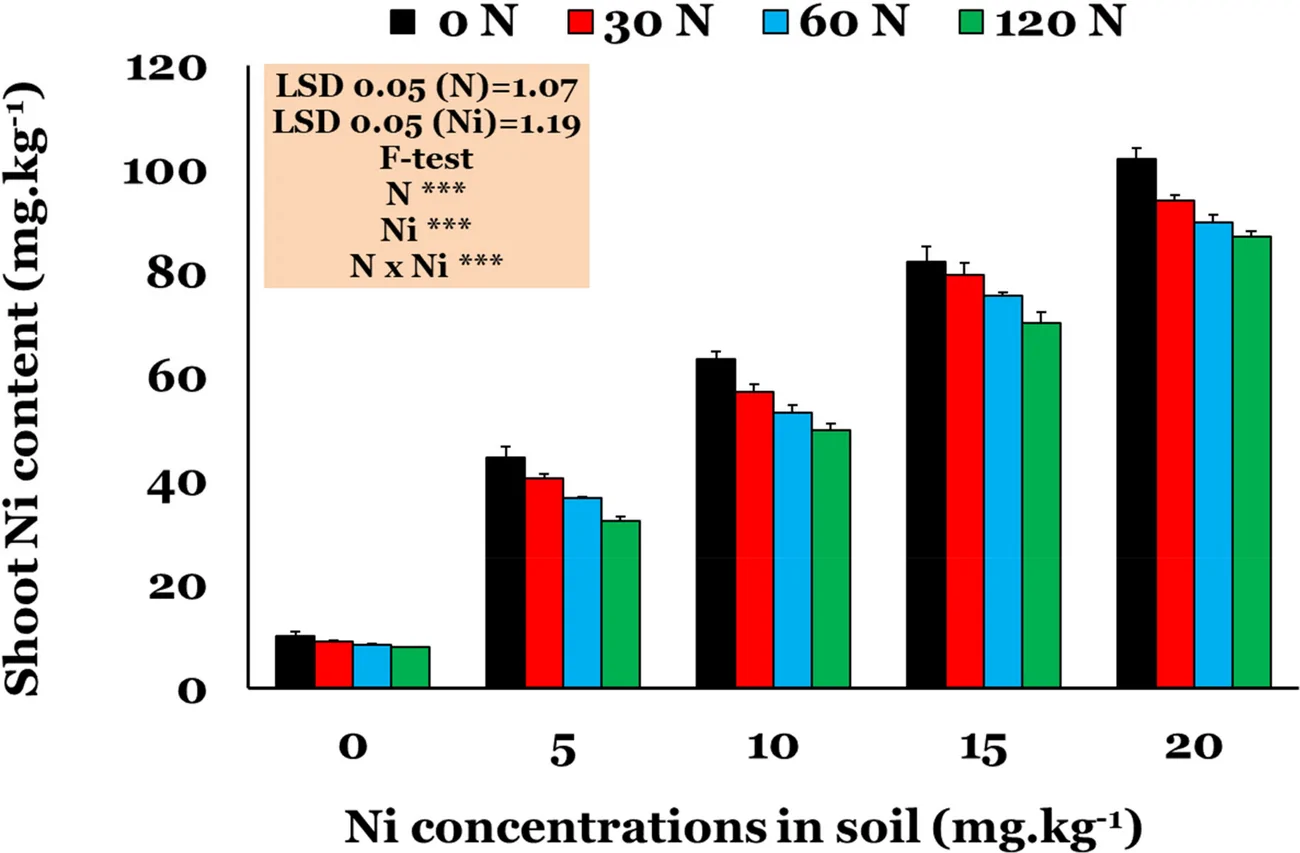
Shoot Ni content in maize plants as influenced by Ni application in soil

This pattern suggests that nitrogen fertilization may reduce Ni shoot content or its measured expression in plant tissues. A likely explanation is the well-documented dilution effect, where increased biomass production under higher nitrogen supply reduces the concentration of absorbed elements by distributing them across greater plant dry matter. In addition, nitrogen may enhance plant growth rate and tissue expansion, further contributing to a reduction in elemental concentration per unit mass. The interaction between nitrogen and nickel is also evident, as the magnitude of Ni-induced increases was highest at low nitrogen levels and gradually reduced as nitrogen increased. At all N levels, the highest values were consistently recorded at 20 mg/kg Ni, confirming that Ni uptake or accumulation is primarily driven by external Ni availability. However, the slope of increase across Ni treatments becomes slightly less pronounced under higher nitrogen levels, suggesting that nitrogen may partially suppress Ni shoot content or translocation. This could be associated with competitive interactions between ammonium/nitrate uptake pathways and metal transport systems in roots, or with improved plant physiological regulation under adequate nitrogen supply. Physiologically, the strong increase in response with Ni addition may reflect enhanced Ni mobility in soil–plant systems and its passive uptake through metal transporters that are not fully selective. At higher concentrations, Ni is readily absorbed and translocated, leading to accumulation in plant tissues. On the other hand, nitrogen fertilization is known to modify rhizosphere chemistry, root membrane transport activity, and overall nutrient balance, which can indirectly influence heavy metal uptake.

The decrease in shoot Ni concentration with increasing N supply may arise from several interacting physiological processes rather than from a simple suppression of Ni uptake. Enhanced N availability generally stimulates leaf expansion, biomass accumulation, and N assimilation; consequently, if biomass production increases faster than net Ni acquisition, the absorbed Ni by shoots becomes distributed over a greater dry-matter pool, producing a lower tissue Ni concentration through a dilution effect [7, 42, 46]. Such nutrient dilution is a recognized consequence of enhanced growth following N fertilization. At the same time, N nutrition can potentially modify Ni acquisition indirectly through changes in rhizosphere chemistry, root development, ion competition, and cellular nutrient-homeostasis pathways. Nickel does not appear to have a highly specific uptake system and can enter roots through relatively poorly selective metal transport systems, including ZIP/IRT-related transporters; therefore, changes in the nutritional status of the plant may alter the balance between Ni influx, intracellular chelation, sequestration, and root-to-shoot transport. Moreover, Ni is closely connected with N metabolism because it is an essential component of urease, meaning that N and Ni nutrition are physiologically interconnected rather than independent processes. Thus, the lower shoot Ni concentration observed under higher N rates should be interpreted primarily as an alteration in Ni concentration and partitioning, with dilution likely contributing substantially, while a direct inhibition of Ni uptake or translocation should only be proposed when supported by corresponding reductions in total Ni uptake or Ni translocation indices. This distinction is particularly important because recent research emphasizes that Ni accumulation is determined by the combined effects of uptake, root sequestration, transport, and plant growth rather than tissue concentration alone [17, 47].

### Soil urease activity

Soil urease activity was significantly affected by both N fertilization and Ni application, as evidenced by the highly significant main effects and N × Ni interaction (*P* < 0.001) (Table 3).

**Table 3:** Urease activity in studied soil treated with different levels of nitrogen and nickel

Urease activity in soil (µg NH₄⁺-*N* g⁻¹ soil h⁻¹)					
*N* levels	Ni levels (mg.kg^-1^)				
	0	5	10	15	20
**0 N**	18 ± 1.2	26 ± 1.8	28 ± 2.0	15 ± 1.1	10 ± 0.8
**30 N**	24 ± 1.6	33 ± 2.2	35 ± 2.4	19 ± 1.3	14 ± 1.0
**60 N**	45 ± 3.0	63 ± 4.1	68 ± 4.5	32 ± 2.2	22 ± 1.5
**120 N**	58 ± 3.8	74 ± 4.9	78 ± 5.1	40 ± 2.7	29 ± 2.0
**LSD**_**0.05**_	*N* = 2.11,** Ni = 1.23**,** N x Ni = 1.02**				
**ANOVA**					
**N**	***				
**Ni**	***				
**N x Ni**	***				

Urease activity increased consistently with increasing N levels from 0 to 120 N under all Ni treatments, indicating enhanced soil biochemical functioning and accelerated nitrogen cycling. The increase in urease activity following N application may be attributed to greater microbial proliferation and increased substrate availability as a possible mechanism. Nitrogen fertilization may stimulate microbial biomass carbon and microbial enzyme synthesis, thereby increasing the capacity of soil microorganisms to hydrolyze urea into ammonium. Enhanced urease activity under higher N rates also suggests the possibility of improved nutrient turnover and a more active soil microbial community. Recent studies indicate that nitrogen management strongly influences urease-mediated N transformations and ultimately affects nitrogen-use efficiency in agricultural systems [48]. The observed increase in soil urease activity following the applied treatments indicates enhanced enzymatic hydrolysis of urea and consequently a potentially faster conversion of urea-N into ammonium (NH₄⁺-N). Urease-mediated hydrolysis represents an important initial step in the transformation of urea-N and can increase the pool of mineral N available for subsequent plant uptake or microbial transformations. However, greater urease activity should not be interpreted as direct evidence of enhanced nitrogen-use efficiency (NUE) or overall N-cycling efficiency, because the fate of the NH₄⁺-N released through urea hydrolysis depends on several concurrent processes, including plant uptake, microbial immobilization, nitrification, NH₃ volatilization, and N leaching or denitrification. In particular, urea hydrolysis can temporarily increase the pH around the hydrolysis site and shift the NH₄⁺/NH₃ equilibrium toward gaseous NH₃, thereby potentially increasing N-loss risk under favorable conditions. Because NH₃ volatilization and other N-loss pathways were not directly quantified in the present study, the higher urease activity observed here should therefore be interpreted as evidence of enhanced urea hydrolysis and accelerated soil N transformation, rather than as conclusive evidence of improved N retention or NUE. Further assessment of plant N recovery together with N-loss pathways would be required to establish whether the stimulation of urease activity ultimately translates into greater N-use efficiency.

Nickel supplementation exhibited a biphasic response. Urease activity increased at low to moderate Ni concentrations (5–10 mg kg⁻¹), but declined sharply at higher Ni concentrations (15–20 mg kg⁻¹). This trend reflects the dual role of Ni as both an essential micronutrient and a potential toxic element. Nickel is an indispensable structural component of urease because the active site of the enzyme contains Ni ions that participate directly in urea hydrolysis. Consequently, moderate Ni availability can enhance urease synthesis and catalytic efficiency in soil microorganisms and plants [39]. The maximum urease activity (78 ± 5.1 µg NH₄⁺-N g⁻¹ soil h⁻¹) was recorded at 120 N combined with 10 mg kg⁻¹ Ni, suggesting a synergistic relationship between adequate nitrogen supply and optimal Ni availability. Under these conditions, microbial populations likely experienced improved nutritional status, resulting in enhanced enzyme production and greater nitrogen mineralization capacity. However, increasing Ni concentrations beyond 10 mg kg⁻¹ significantly reduced urease activity. At 120 N, urease activity declined from 78 to 40 and 29 µg NH₄⁺-N g⁻¹ soil h⁻¹ at 15 and 20 mg kg⁻¹ Ni, respectively. This reduction may be due to Ni toxicity, which disrupts microbial metabolism, damages cellular membranes, induces oxidative stress, and suppresses enzyme synthesis. Excessive Ni may also interfere with nutrient uptake and alter microbial community composition. Recent reviews describe how elevated Ni concentrations negatively affect soil microorganisms and ecosystem functioning [49]. The significant N × Ni interaction further indicates that the influence of Ni depended on the N fertilization level. Nitrogen amplified the beneficial effects of moderate Ni concentrations but could not overcome the inhibitory effects of excessive Ni. This interaction demonstrates that balanced nutrient management is necessary because micronutrient benefits can rapidly transition into toxicity when threshold concentrations are exceeded. The observed treatment responses also indicate that urease activity can serve as a sensitive indicator of soil health. Soil urease is among the earliest biochemical properties to respond to nutrient imbalance and heavy metal stress, making it useful for monitoring soil quality and sustainability. Recent reviews emphasize that heavy metals substantially alter nitrogen-cycling enzymes and their associated microbial functions [50]. The LSD values (*N* = 2.11, Ni = 1.23, N × Ni = 1.02) demonstrate that relatively small changes among treatments were statistically significant, indicating a strong treatment effect and low experimental variability. The highly significant ANOVA results (*P* < 0.001) confirm that both factors independently and interactively regulated urease activity. Overall, moderate Ni supplementation (5–10 mg kg⁻¹) together with higher N application improved soil urease activity and likely enhanced nitrogen cycling efficiency. Conversely, excessive Ni (15–20 mg kg⁻¹) suppressed urease activity due to metal toxicity. Therefore, optimizing both N and Ni management may improve soil biological fertility while avoiding adverse effects associated with heavy metal accumulation. The study of Sakin et al., [51] may be good indicators of enzyme activities, especially dehydrogenase, catalase, and urease, for soil health and quality, chemical degradation and restoration processes, and ecosystem functioning in soils contaminated or to be contaminated with heavy metals. It shows that the activities of these enzymes are very sensitive and can decrease rapidly in case of high concentrations of heavy metals. The study of Cun et al., [52] revealed that soil enzyme activities increased markedly across all Mo–N treatments, reflecting enhanced soil biochemical functioning. The Mo–N interaction positively influenced cotton physiology by enhancing photosynthetic performance, root development, nitrogen assimilation, and enzymatic antioxidant activity. Mo applications combined with appropriate N levels improved plant performance and offer a promising strategy for strengthening cotton productivity under diverse soil conditions.

### Estimated crude protein in maize plants

The present study demonstrated that estimated crude protein (CP) content was significantly influenced by nitrogen (N) fertilization, nickel (Ni) application, and their interaction, as indicated by the highly significant ANOVA results (*P* < 0.001) (Table 4).

**Table 4:** Estimated crude protein percentage in shoot of maize plant grown in the studied soil treated with different levels of nitrogen and nickel

Estimated crude protein (%)					
*N* levels	Ni levels (mg.kg^-1^)				
	0	5	10	15	20
**0 N**	6.98 ± 0.31	10.73 ± 0.45	11.54 ± 0.52	5.92 ± 0.26	4.13 ± 0.21
**30 N**	10.08 ± 0.48	14.29 ± 0.63	15.25 ± 0.69	7.94 ± 0.36	5.88 ± 0.29
**60 N**	20.58 ± 0.92	29.63 ± 1.28	32.10 ± 1.41	14.77 ± 0.67	9.56 ± 0.44
**120 N**	27.04 ± 1.18	35.31 ± 1.53	37.06 ± 1.62	18.92 ± 0.84	14.17 ± 0.63
**LSD**_**0.05**_	*N* = 2.66,** Ni = 1.55**,** N x Ni = 1.31**				
**ANOVA**					
**N**	***				
**Ni**	***				
**N x Ni**	***				

Estimated crude protein increased progressively with increasing N levels, while Ni exerted both stimulatory and inhibitory effects depending on its concentration. The highest estimated crude protein content (37.06 ± 1.62%) was recorded at 120 N combined with 10 mg kg⁻¹ Ni, whereas the lowest value (4.13 ± 0.21%) occurred at 0 N with 20 mg kg⁻¹ Ni. These findings suggest that balanced N and Ni nutrition can synergistically enhance estimated protein accumulation, whereas excessive Ni levels become detrimental to plant metabolism. The high value of 37.06% at 5.93% total N represents an N-derived crude protein equivalent and may include non-protein N compounds, such as accumulated nitrate and other soluble N forms, particularly in actively growing vegetative maize tissue. Similar findings have been widely reported, where improved nitrogen utilization increased amino acid biosynthesis, photosynthetic activity, and protein accumulation in crops [3].

Nitrogen fertilization was the primary factor controlling estimated crude protein accumulation. Nitrogen is an essential constituent of amino acids, peptides, proteins, nucleic acids, chlorophyll molecules, enzymes, and several secondary metabolites. Therefore, increasing N availability directly promotes protein synthesis through enhanced nitrogen assimilation [53, 54]. In the present study, CP increased substantially from 6.98% under 0 N to 27.04% under 120 N in the absence of Ni supplementation. This response indicates that adequate N availability improved nitrogen uptake, assimilation, and incorporation into amino acids and proteins. Nitrogen fertilization also stimulates vegetative growth, leaf area expansion, and chlorophyll synthesis, thereby increasing photosynthetic capacity and the availability of carbon skeletons necessary for amino acid production. The observed increase in estimated crude protein with higher N rates may also be attributed to enhanced activity of enzymes involved in nitrogen metabolism. Nitrogen absorbed by plants is converted through a coordinated enzymatic pathway involving nitrate reductase, nitrite reductase, glutamine synthetase, glutamate synthase, and glutamate dehydrogenase. These enzymes convert inorganic nitrogen into organic compounds that serve as precursors for protein biosynthesis. Greater nitrogen availability accelerates these metabolic pathways, ultimately increasing the concentration of proteins in plant tissues. Similar findings have been reported in numerous crop studies, where increased N fertilization significantly improved forage quality, protein concentration, and nutritional value [3].

Nickel supplementation exhibited a dose-dependent effect on estimated crude protein accumulation. Moderate Ni concentrations (5–10 mg kg⁻¹) significantly enhanced estimated crude protein content at all N levels, whereas higher Ni concentrations (15–20 mg kg⁻¹) reduced protein accumulation. This dual response reflects the essential yet potentially toxic nature of Ni in biological systems. Nickel is recognized as an essential micronutrient because it is a structural component of urease, the enzyme responsible for hydrolyzing urea into ammonia and carbon dioxide. Adequate Ni availability facilitates efficient nitrogen recycling and improves soil nitrogen transformations [39], thereby promoting amino acid and protein synthesis. The increase from 27.04% to 37.06% crude protein at 120 N following the application of 10 mg kg⁻¹ Ni clearly demonstrates this beneficial effect. The positive effect of moderate Ni concentrations may also be associated with improvements in soil biochemical processes and rhizosphere microbial activity. Nickel can indirectly enhance nutrient transformations by stimulating urease activity, thereby increasing ammonium availability for plant uptake. Enhanced nitrogen availability subsequently increases amino acid biosynthesis and protein accumulation. In addition, adequate Ni levels may improve root growth and nutrient absorption efficiency, allowing plants to utilize applied nitrogen more effectively. Consequently, the combination of sufficient N fertilization and moderate Ni supplementation creates favorable conditions for maximizing protein synthesis. The beneficial effect of moderate Ni concentrations may also be associated with enhanced root function and nutrient acquisition. Improved nutrient uptake may increase nitrogen translocation from roots to shoots, facilitating amino acid synthesis and protein accumulation. Moreover, optimal Ni concentrations may support microbial activity in the rhizosphere, indirectly improving nutrient availability and plant nutritional status. These mechanisms collectively explain the superior performance observed at 5–10 mg kg⁻¹ Ni. Rabinovich et al. [39] reported that improving Ni bioavailability can enhance urease activity in both plants and soil microorganisms, thereby increasing urea-N utilization efficiency and supporting sustainable crop production. However, excessive Ni concentrations caused a substantial decline in estimated crude protein accumulation. At 120 N, crude protein decreased from 37.06% at 10 mg kg⁻¹ Ni to 18.92% and 14.17% at 15 and 20 mg kg⁻¹ Ni, respectively. This reduction suggests that the threshold between micronutrient sufficiency and toxicity had been exceeded. Nickel toxicity may disrupt several physiological and biochemical processes, including photosynthesis, respiration, membrane stability, and nutrient uptake as possible mechanisms. Excess Ni may induce oxidative stress through excessive generation of reactive oxygen species (ROS), which damage proteins, lipids, nucleic acids, and cellular membranes. Oxidative damage subsequently suppresses protein synthesis and accelerates protein degradation [39]. Another mechanism underlying the reduction in crude protein at elevated Ni concentrations is nutrient antagonism. Excessive Ni can interfere with the uptake and translocation of essential nutrients such as iron, magnesium, zinc, calcium, and manganese. These nutrients are required for chlorophyll formation, enzymatic activation, and metabolic regulation. Their deficiency may impair photosynthetic performance and reduce the supply of energy and carbon skeletons needed for amino acid synthesis. Furthermore, high Ni concentrations may inhibit key enzymes involved in nitrogen assimilation, thereby reducing the conversion efficiency of absorbed nitrogen into proteins. The highly significant N × Ni interaction indicates that the effect of Ni was strongly dependent on nitrogen availability. At low N levels, the beneficial effect of moderate Ni supplementation was relatively limited because nitrogen availability itself restricted protein synthesis. However, under higher N fertilization, plants were able to capitalize on the improved nitrogen metabolism associated with optimal Ni concentrations. This synergistic relationship explains the superior performance observed at 120 N combined with 10 mg kg⁻¹ Ni. Nevertheless, excessive Ni reduced crude protein even under abundant N conditions, indicating that metal toxicity can override the positive effects of increased nitrogen supply. The low LSD values (*N* = 2.66, Ni = 1.55, and N × Ni = 1.31) further confirm that the treatments exerted strong and consistent effects on crude protein accumulation. The highly significant ANOVA results (*P* < 0.001) demonstrate that both nutrient management and micronutrient balance are critical determinants of plant nutritional quality. These findings emphasize the importance of integrated nutrient management strategies that optimize N availability while maintaining Ni within beneficial ranges. Overall, the study suggests that moderate Ni supplementation (5–10 mg kg⁻¹) can improve the efficiency of nitrogen utilization and enhance crude protein accumulation, particularly under adequate N fertilization. However, excessive Ni application should be avoided because of its adverse effects on plant metabolism and nutritional quality. Therefore, maintaining an optimal balance between macronutrients and micronutrients is essential for maximizing crop productivity and improving protein quality.

## Conclusion

The present study demonstrated that nitrogen (N) and nickel (Ni) significantly influenced maize productivity, N uptake, soil urease activity, and N-derived crude protein equivalent in calcareous soil. Nitrogen was the primary driver of plant growth and N accumulation, whereas Ni showed beneficial effects only within a limited concentration range. Moderate Ni application (5–10 mg kg⁻¹ soil) generally improved plant performance, particularly under adequate N supply, while higher rates (15–20 mg kg⁻¹ soil) reduced growth and N-related responses, confirming a biphasic Ni dose response. Under the present experimental conditions, 120 mg N kg⁻¹ soil combined with 10 mg Ni kg⁻¹ soil produced the most favorable responses; however, this should not be regarded as a universal field recommendation.

Crude protein values were calculated from total N using a factor of 6.25 and therefore represent an N-derived crude protein equivalent rather than direct measurements of true protein accumulation. Furthermore, because post-harvest soil Ni and its available fraction were not assessed, the persistence and environmental fate of applied Ni remain uncertain. The controlled greenhouse conditions also limit direct extrapolation to field-scale production. Long-term field studies incorporating residual soil Ni measurements and direct assessment of protein-bound N are therefore needed to validate the agronomic benefits and environmental safety of Ni application.

## Data Availability

Availability of data and materials: Data of this study will be available when it is requested.
